# A rare case of giant atypical parathyroid tumor presenting as a parathyroid adenoma – discussion of intraoperative challenges

**DOI:** 10.1093/jscr/rjag731

**Published:** 2026-08-23

**Authors:** Mehnaaz Mohammed, Sharmila Raju, Neda Salami, So Un Kim, Angel Guan, Aldin Malkoc, John Magpayo, Judi Anne Ramiscal

**Affiliations:** Department of General Surgery, Arrowhead Regional Medical Center, 400 N. Pepper Ave., Colton, CA 92324, United States; Department of General Surgery, Arrowhead Regional Medical Center, 400 N. Pepper Ave., Colton, CA 92324, United States; Department of General Surgery, Arrowhead Regional Medical Center, 400 N. Pepper Ave., Colton, CA 92324, United States; Department of General Surgery, Arrowhead Regional Medical Center, 400 N. Pepper Ave., Colton, CA 92324, United States; Department of General Surgery, Arrowhead Regional Medical Center, 400 N. Pepper Ave., Colton, CA 92324, United States; Department of General Surgery, Arrowhead Regional Medical Center, 400 N. Pepper Ave., Colton, CA 92324, United States; Department of Laboratory Medicine, Arrowhead Regional Medical Center, 400 N. Pepper Ave., Colton, CA 92324, United States; Department of General Surgery, Arrowhead Regional Medical Center, 400 N. Pepper Ave., Colton, CA 92324, United States

**Keywords:** atypical parathyroid tumor, parathyroid adenoma, hyperparathyroidism, parathyroid

## Abstract

Atypical parathyroid tumors are variants of parathyroid adenomas that may present with features of parathyroid carcinoma due to their size, biochemical profile, and atypical features that are typically associated with carcinomas. We report a case of a 75-year-old female presenting with chest pain, hypercalcemia, and severely elevated parathyroid hormone level. Intraoperatively, a 40-g gland was found displacing adjacent structures, raising concern for malignancy. The parathyroid lesion was determined to be an atypical parathyroid tumor. This case highlights the diagnostic challenge in distinguishing atypical parathyroid tumors and highlights the importance of advanced imaging in surgical planning and management of atypical presentations of primary hyperparathyroidism.

## Introduction

Primary hyperparathyroidism is most commonly caused by parathyroid adenomas, which typically present with hypercalcemia and modest gland enlargement. These adenomas have a median weight of ~500 mg, compared to normal glands at about 62 mg [1].

Atypical parathyroid tumors represent a distinct entity, characterized by histologic features concerning for carcinoma without definitive evidence of invasion [2]. Distinguishing large benign adenomas from carcinoma is critical, as it directly impacts surgical strategy and postoperative management. We present a rare case of an atypical parathyroid tumor initially concerning for carcinoma. The patient presented with symptomatic hypercalcemia and elevated parathyroid hormone (PTH), with imaging suggesting a large left-sided adenoma. Intraoperatively, the lesion’s size and proximity to the subclavian vessels heightened concern for malignancy. However, after excision, the mass appeared well-circumscribed without invasion. Final pathology confirmed an atypical parathyroid tumor, highlighting the diagnostic challenges and the importance of integrating clinical, biochemical, and intraoperative findings.

## Case presentation

**Figure 1:**
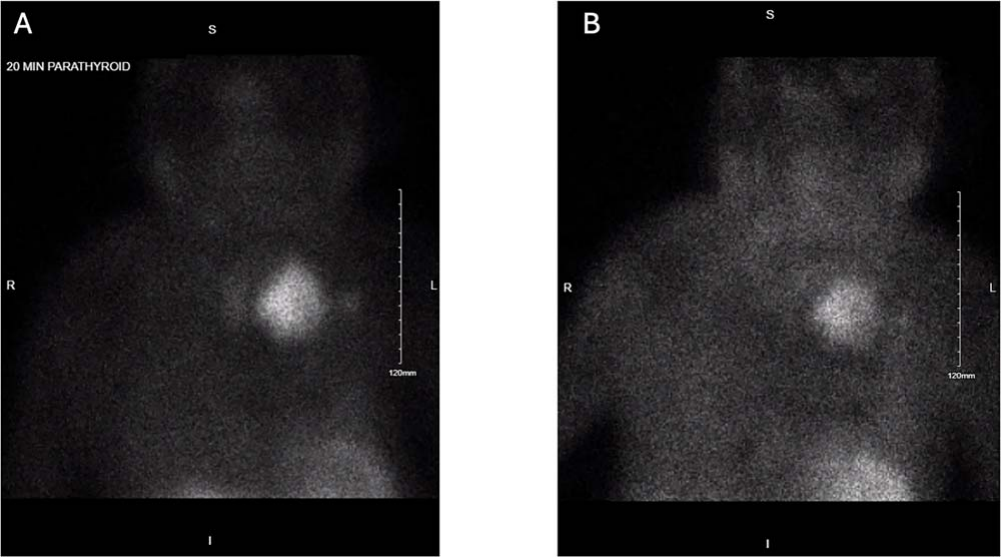
(A) Increased immediate areas of uptake in the region of the left thyroid in the 20-minute images. (B) No washout in 3-hour delayed images, suggestive of left parathyroid adenoma.

A 75-year-old female with a history of cholecystectomy, nephrolithiasis, and chronic kidney disease presented to the emergency department with acute-onset palpitations and chest pain radiating to the left arm. In the emergency department, the patient was found to have a body mass index of 13, attributed to malnutrition, and was hypertensive and bradycardic. Initial labs revealed significant hypercalcemia to 14.2 mg/dl and elevated PTH to 719 pg/ml. Electrocardiogram was concerning for STEMI, but subsequent urgent cardiac catheterization demonstrated no coronary obstruction. Imaging revealed a 0.7 cm left proximal ureteropelvic junction stone with severe left hydronephrosis and multiple right renal calculi with moderate hydronephrosis. The patient was treated with normal saline for the symptomatic hypercalcemia. A Tc-99 m sestamibi scan was performed, demonstrating a large left-sided parathyroid adenoma (Fig. 1).

**Figure 2:**
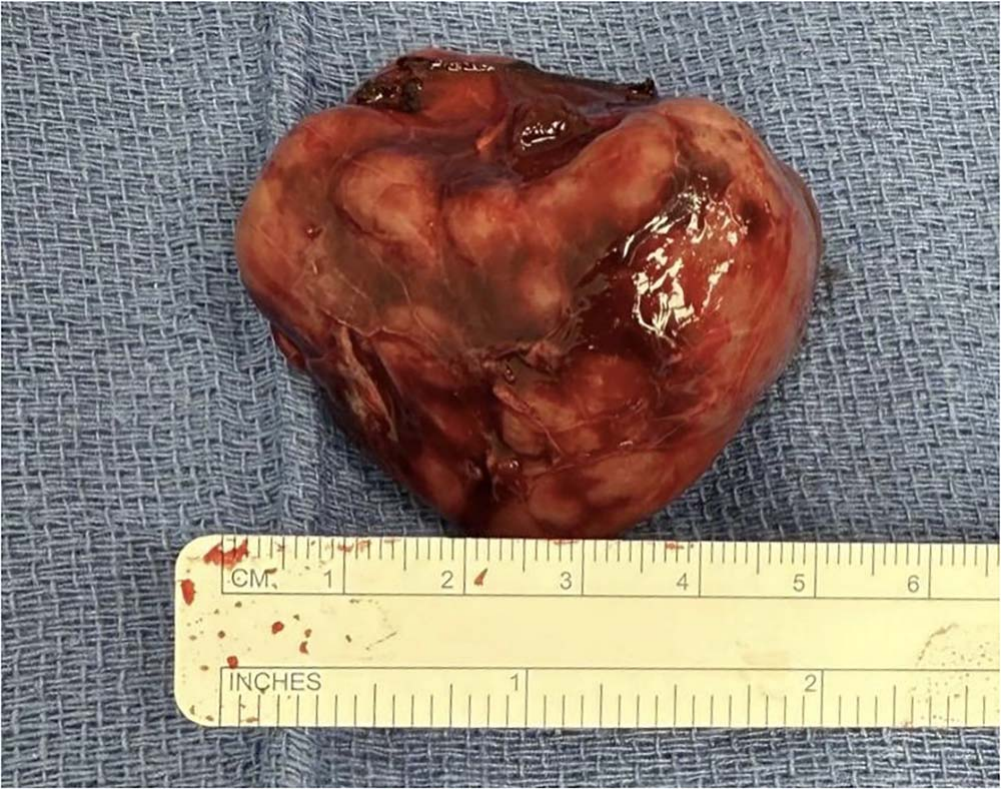
Intraoperative image of parathyroid gland after excision.

The patient underwent successful inpatient parathyroidectomy. Intraoperatively, the large parathyroid mass was identified near subclavian vessels and required meticulous dissection during resection (Fig. 2). Intraoperative PTH level at the beginning of the surgery was 1745 pg/ml. A 40-g, 6.5 cm by 5 cm by 4 cm large parathyroid mass was then excised. PTH levels dropped to 365 pg/ml within 10 minutes.

Final in-house pathology demonstrated large encapsulated parathyroid tissue, consistent with parathyroid adenoma. Postoperative labs showed normalization of calcium and PTH within six hours. The patient was discharged three days after surgery with calcium supplementation.

**Figure 3:**
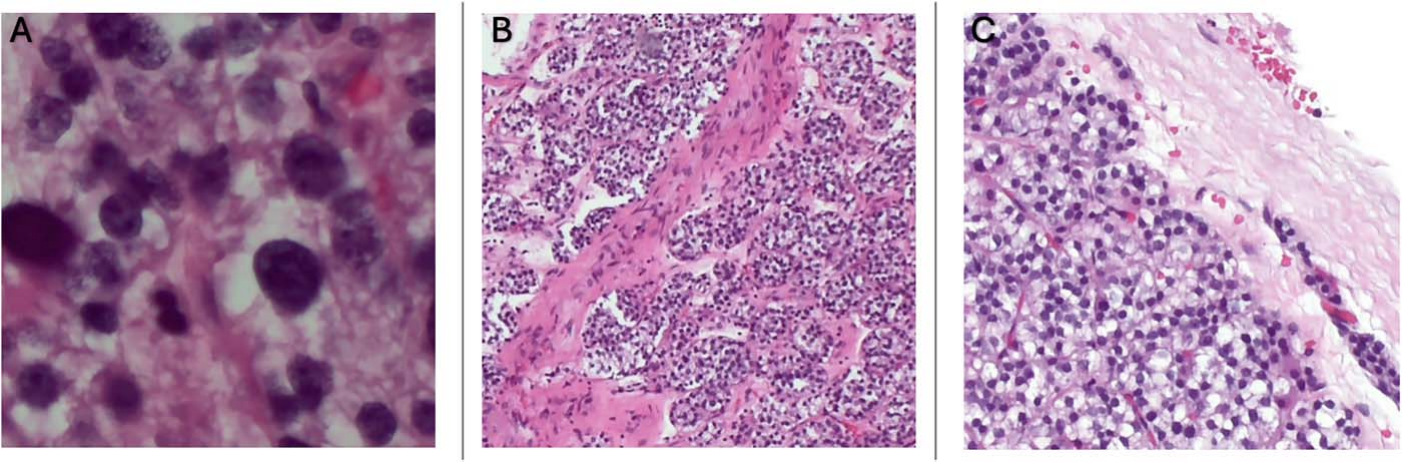
(A) Patient’s parathyroid gland demonstrating nuclear atypia, a feature commonly seen in parathyroid carcinoma. (B) Parathyroid gland demonstrating broad fibrous bands, another feature associated with parathyroid carcinoma. (C) Parathyroid gland demonstrating intact capsule with no invasion, thus lacking the diagnostic feature of parathyroid carcinoma.

The patient presented to the hospital 2 weeks after surgery with symptomatic hypocalcemia and an elevated PTH of 151, which continued to remain elevated despite calcium supplementation. Due to concern for malignancy, outside expert pathologic consultation was obtained, which showed a well-circumscribed parathyroid neoplasm with dense fibrous bands and focal nuclear atypia (Fig. 3). However, there was no evidence of capsular invasion with extension into adjacent soft tissue, no vascular or lymphatic invasion, no perineural invasion, and no metastatic disease identified. Immunohistochemical analysis demonstrated a Ki-67 proliferation index of five percent with retained parafibromin expression. The parathyroid mass was determined to be an atypical parathyroid tumor.

## Discussion

Atypical parathyroid tumors are variants of parathyroid adenomas that exhibit concerning histologic features suggestive of malignant potential but lack definitive evidence of invasion or metastasis, and therefore do not meet criteria for parathyroid carcinoma. Parathyroid carcinoma can present similarly, with severe hypercalcemia and significantly elevated PTH, making preoperative differentiation challenging. This distinction is critical, as en bloc resection during the initial operation is associated with improved outcomes in parathyroid carcinoma [3].

Histopathologic evaluation revealed features atypical for parathyroid adenomas, including nuclear atypia and broad fibrous bands. However, there was no evidence of capsular or vascular invasion to support a diagnosis of parathyroid carcinoma. Therefore, according to the 2022 WHO Classification of Endocrine and Neuroendocrine Tumors, the lesion was classified as an atypical parathyroid tumor [4].

Atypical parathyroid tumors have uncertain malignant potential and require closer postoperative surveillance. The 2022 WHO classification recommends immunohistochemical analysis for risk stratification for future recurrence [4]. This patient’s parathyroid mass had a Ki-67 proliferation index of five percent and retained nuclear parafibromin expression. Elevated Ki-67 indices of greater than 5% and loss of parafibromin staining are more commonly associated with parathyroid carcinoma and increased recurrence risk [4]. Thus, this patient’s immunohistochemical profile suggests a lower risk of recurrence compared with tumors demonstrating these high-risk features, although continued surveillance remains warranted given the tumor’s atypical nature.

An alternate imaging modality, such as four-dimensional computed tomography (4D CT), may better visualize findings suggestive of local invasion than traditional sestamibi imaging [5, 6]. Preoperative use of advanced imaging could improve localization, refine surgical strategy, and potentially impact outcomes in cases with concern for atypical or malignant pathology [7]. In this case, lack of 4D CT limited the ability to fully characterize the lesion, contributing to increased operative complexity and time.

Atypical parathyroid tumors are rare and can be challenging to diagnose preoperatively. In this case, a 75-year-old female initially suspected to have a large parathyroid adenoma was ultimately found to have an atypical parathyroid tumor. This case highlights the importance of maintaining suspicion for atypical or malignant pathology in patients with severe hyperparathyroidism and suggests that advanced preoperative imaging, such as 4D CT, may improve lesion characterization, facilitate operative planning, and help anticipate increased surgical complexity.
